# Using weighted gene co-expression network analysis to identify key genes related to preeclampsia

**DOI:** 10.3389/fimmu.2025.1569591

**Published:** 2025-03-26

**Authors:** Xinyang Shen, Zhirui Zeng, Lijia Xie, Xiaojing Yue, Zhijian Wang

**Affiliations:** ^1^ Department of Obstetrics and Gynecology, Nanfang Hospital, Southern Medical University, Guangzhou, Guangdong, China; ^2^ Department of Physiology, School of Basic Medical Sciences; Guizhou Provincial Key Laboratory of Pathogenesis and Drug Research on Common Chronic Diseases, Guizhou Medical Universityu, Guiyang, Guizho, China; ^3^ Department of Obstetrics and Gynecology, The Tenth Affiliated Hospital of Southern Medical University (Dongguan People’s Hospital), Guizhou, China; ^4^ Department of Obstetrics and Gynecology, Guangdong Provincial Key Laboratory of Major Obstetric Diseases, Guangdong Provincial Clinical Research Center for Obstetrics and Gynecology, Guangdong-Hong Kong-Macao Greater Bay Area Higher Education Joint Laboratory of Maternal-Fetal Medicine, The Third Affiliated Hospital, Guangzhou Medical University, Guangzhou, China

**Keywords:** preeclampsia, placenta, WGCNA, GSEA, immunohistochemistry

## Abstract

**Introduction:**

The pathogenesis of preeclampsia remains unclear, highlighting the need for the creation of dependable biomarkers. This study aimed to pinpoint genetic risk factors linked to preeclampsia through the utilization of weighted gene co-expression network analysis (WGCNA).

**Methods:**

A gene expression profile dataset from the placentas of patients with preeclampsia was acquired from the Gene Expression Omnibus (GEO) database and employed as a discovery cohort to construct a WGCNA network. Functional enrichment analysis, pathway analysis, and the construction of protein–protein interaction (PPI) networks were performed on core genes within these modules to pinpoint hub genes. The GSE25906 dataset was utilized as a validation cohort to evaluate the diagnostic significance of the hub genes. Immunohistochemistry assays were employed to validate the protein expression levels of these genes in placental tissues from both preeclampsia and control groups.

**Results:**

Through WGCNA, 33 co-expression modules were identified, with 4 modules significantly associated with multiple clinical traits (≥3). Among these, 75 core genes were highlighted, predominantly enriched in pathways related to the adaptive immune response and platelet activation. Notably, *TYROBP, PLEK, LCP2, HCK, and ITGAM* emerged as hub genes with high PPI network scores and strong diagnostic potential, all prominently associated with immunity-related pathways. Protein expression analysis revealed that these genes were downregulated in placental tissues from preeclampsia patients compared to healthy controls.

**Discussions:**

*TYROBP, PLEK, LCP2, HCK, and ITGAM* are closely linked to preeclampsia and hold promise as potential biomarkers for its diagnosis and for advancing the understanding of its pathogenesis.

## Introduction

Preeclampsia is a common obstetric condition affecting 5–7% of pregnant women, resulting in over 70,000 maternal deaths and 500,000 fetal deaths annually (1). It is defined by elevated blood pressure (BP, systolic >140 mmHg, diastolic >90 mmHg) after 20 weeks of pregnancy, accompanied by proteinuria or severe complications. Numerous studies have shown that preeclampsia significantly increases the long-term risk of cardiovascular disease for both the mother and fetus (1, 2). Furthermore, it raises the likelihood of mental and neurological disorders in offspring due to premature birth (3). While the exact etiology of preeclampsia remains unclear, research has demonstrated that abnormal placental formation and impaired placental vascularization play key roles in its pathophysiology (4). Identifying genetic risk factors associated with preeclampsia is essential for understanding its pathogenesis and could provide valuable biomarkers for prediction and early diagnosis. Genetic factors are increasingly recognized as critical contributors to preeclampsia, particularly in the context of trophoblast dysfunction. Trophoblasts, which are essential for placental development and fetal-maternal communication, exhibit altered gene expression patterns in preeclampsia. These genetic changes can disrupt trophoblast invasion, spiral artery remodeling, and placental angiogenesis, leading to placental insufficiency and the clinical manifestations of preeclampsia.

Bioinformatics analysis of placental gene expression profiles is a powerful approach for identifying genes involved in the progression of preeclampsia (5). WGCNA is an advanced method that clusters genes associated with clinical traits using sophisticated algorithms. These gene clusters, or modules, are then correlated with specific clinical characteristics (6). Notably, WGCNA has been shown to identify immune-related molecular markers linked to immune cell infiltration in preeclampsia (7).

In this study, we analyzed the GSE75010 dataset, which includes gene expression profiles from the placentas of 80 preeclampsia patients and 77 healthy pregnant women. With its comprehensive clinical data, this dataset is well-suited for investigating gene-cluster associations with clinical traits. Core genes were identified and further analyzed using Kyoto Encyclopedia of Genes and Genomes (KEGG) pathway enrichment analysis and functional annotation. To explore the biological characteristics of these core genes, we employed Gene Set Enrichment Analysis (GSEA), which integrates datasets and gene expression levels to predict biological functions. Additionally, the protein expression of five core genes was validated through immunohistochemistry in placental tissue sections from 18 preeclampsia patients and 15 healthy controls. Our study distinguishes itself by focusing on a novel aspect of the dataset exploring the role of a newly identified gene network in disease progression, which has not been investigated in earlier analyses. By employing WGCNA, a well-established and effective method, we ensure consistency with prior studies while uncovering new insights. Our work not only builds on existing findings but also advances the field by integrating additional validation experiments and proposing potential biomarkers for its diagnosis and for advancing the understanding of its pathogenesis.

## Materials and methods

### Data collection and processing

Gene expression profile datasets GSE75010 and GSE25906 were downloaded from the Gene Expression Omnibus (GEO) database (http://www.ncbi.nlm.nih.gov/geo) (8). GSE75010 served as the primary cohort for discovery, while GSE25906 was utilized as the validation cohort. The GSE75010 dataset comprised gene expression profiles from 80 preeclampsia placentas and 77 non-preeclampsia placentas, generated using the GPL6244 platform (Affymetrix Human Gene 1.0 ST Array). The GSE25906 dataset, generated with the Illumina Human-6 v2.0 expression beadchip on the GPL6102 platform, included gene expression profiles from 23 preeclampsia and 37 normal control placental samples.

Probe-to-Gene Annotation: Probe names in the GSE75010 dataset were converted to gene names using the platform annotation file. For genes with multiple corresponding probes, the average expression value of these probes was calculated to represent the gene expression level. This step ensured that each gene was uniquely represented in the dataset. Quality Control: Raw expression data from both datasets were subjected to quality control checks, including the removal of probes with missing or low expression values across samples. Normalization: The datasets were normalized to correct for batch effects and technical variations using the limma package in R. This step ensured comparability between samples within and across datasets. Gene Filtering: Genes with low expression or minimal variability across samples were filtered out to reduce noise. Only genes with detectable expression in at least 80% of the samples were retained for further analysis. Final Annotation: After processing, a total of 21,045 genes were meticulously annotated and retained for downstream analysis.

### WGCNA in R software

Before conducting WGCNA, the gene expression profiles of 80 preeclampsia placentas were validated for quality using the “goodSamplesGenes” function in R software (version 3.6.3). A mean FPKM threshold of 0.5 was applied to filter out low-expression genes. Pearson correlation analysis was utilized to cluster the samples and identify outliers, with an outlier threshold set at 60, resulting in the exclusion of one sample (GSM1940547). Thus, 79 preeclampsia placenta samples, containing 21,045 genes, along with clinical characteristics such as age, body mass index (BMI), nulliparity, previous hypertension, mean uterine perfusion index (PI), mean umbilical PI, maximum systolic BP, maximum diastolic BP, proteinuria, delivery mode, gestational days, infant gender, newborn weight z-score, neonatal intensive care unit (NICU) transfer, placental weight z-score, and umbilical cord diameter, were included for WGCNA.

The WGCNA analysis was performed using the R package “WGCNA” (https://www.r-project.org/; version 3.6.2). Gene co-expression relationships were assessed through Pearson’s correlation test, leading to the construction of a similarity matrix. A suitable soft-thresholding power was determined to ensure scale independence (>0.85) and a mean connectivity of approximately 0. The adjacency matrix was then transformed into a topological overlap matrix (TOM), and genes were clustered using the hclust function based on a TOM-based dissimilarity measure (1-TOM). To identify gene modules, the dynamic tree cut method was applied, with a minimum module size set at 30 genes.

### Identification of key clinically significant gene modules and module core genes

The associations between gene modules and clinical traits were examined, focusing on traits such as maternal age, BMI, nulliparity, previous hypertension, mean uterine PI, mean umbilical PI, maximum systolic BP, maximum diastolic BP, proteinuria, mode of delivery, gestational days, infant sex, newborn weight z-score, NICU transfer, placental weight z-score, and umbilical cord diameter. Clinically significant gene modules were established based on the criteria of a correlation score (cor) > 0.3 and a P value < 0.05. Key clinically significant gene modules were identified if they were significant for more than three clinical traits. Subsequently, the module membership (MM) of each gene within these clinically significant modules was calculated, with genes having MM scores ≥ 0.8 designated as module core genes.

### Functional enrichment and KEGG pathway analysis of module genes

All core genes within the module were submitted to DAVID 6.8 (9) (http://david-d.ncifcrf.gov/) for Gene Ontology (GO) functional annotation, which encompasses biological processes, molecular functions and cellular components. KEGG enrichment pathway analysis was performed using Kobas 3.0 (10) (http://kobas.cbi.pku.edu.cn/). Results meeting the criteria for an adjusted P-value < 0.05 were visualized using R software. The following steps were undertaken for the functional enrichment and KEGG pathway analysis of the module genes:

### Construction of protein-protein interaction networks and identification of hub genes

STRNG (11) (version 11.0; https://string-db.org/) was employed to predict interactions among the core genes within the module. The constructed PPI network was then visualized using Cytoscape software (version: 3.7.2; https://cytoscape.org/). Using R software, the associations between genes were quantified, with the count of these associations serving as an indicator of gene connectivity. Hub genes, which are pivotal in the network, were identified by selecting genes with degree scores within the top 10% for further in-depth analysis.

### Validation of hub gene expression

The expression levels of hub genes were assessed in 79 preeclampsia placentas and 77 non-preeclampsia placentas from the GSE75010 gene expression profile dataset, using GraphPad Prism (version 7.0; GraphPad Software, Inc.). Data were presented as mean ± standard deviation. Statistical analysis was conducted using an unpaired independent t-test, with a significance threshold set at P < 0.05.

### Diagnostic assessment of hub genes

The GSE75010 dataset was employed to validate the diagnostic performance of hub genes through Receiver Operating Characteristic (ROC) curve analysis. This dataset comprised 80 preeclampsia placentas and 77 non-preeclampsia placentas. ROC curves were generated using SPSS 22.0 (IBM Corp.), and genes were considered to possess significant diagnostic value when the area under the ROC curve (AUC) exceeded 0.6. Following this, the GSE25906 dataset was utilized as a validation cohort for sensitivity and specificity analysis via ROC curve assessments, employing the same methodology. This approach allowed for a comprehensive evaluation of gene significance across multiple datasets.

### GSEA

GSEA was conducted to investigate the potential roles of hub genes in preeclampsia. In the GSE75010 dataset, 80 preeclampsia samples were divided into two groups—high-expression and low-expression—based on the median levels of hub gene expression. The c2.cp.kegg.v7.1.symbols.gmt dataset was used to assess significant expression differences between these groups. Significance was defined by a P value < 0.05 and a normalized enrichment score (NES) > 1.5.

### Ethical considerations regarding tissue collection

Human placental tissues were obtained and used for this study following written informed consent from patients and approval by the Clinical Ethics Management Committee of Nanfang Hospital of Southern Medical University (NFEC-2019-133, July 26, 2019). Between February 2020 and May 2020, a total of 15 normal placental tissues and 18 preeclamptic placental tissues were collected. Women with a history of smoking, alcohol consumption, multiple gestations, or other complicating conditions were excluded from the study.

### Immunohistochemical staining

All placental tissues were promptly collected within 30 minutes post-delivery and fixed in paraformaldehyde at room temperature for at least 24 hours. After gradual dehydration and paraffin embedding, the tissues were sectioned into 4-μm-thick slices. The sections were then heated at 60°C for one hour, dewaxed with xylene at room temperature, and rehydrated using graded ethanol concentrations (100%, 80%, 60%, and 40%). Antigen retrieval was performed using 100 mmol/L sodium citrate, followed by treatment with 3% hydrogen peroxide (H_2_O_2_) for 20 minutes and 3% bovine serum albumin for 30 minutes at room temperature.

All samples were exposed to primary antibodies for 12 hours at 4°C. After rinsing with PBS, the slides were incubated with horseradish peroxidase (HRP)-labeled goat anti-mouse and rabbit secondary antibodies at room temperature for 2 hours. Imaging was conducted using a fluorescence microscope (Olympus BX51). Immunohistochemistry results were assessed by multiplying the staining intensity by the percentage of positive cells. Staining intensity was graded as follows: 0 (no staining), 1 (+), 2 (++), and 3 (+++), while the proportion of positive cells was scored as: 0 (0-1%), 1 (1-33%), 2 (34-66%), and 3 (67-100%). The antibodies employed in this study were sourced as follows: Anti-TYROBP (DF7316, Affinity Biosciences); Anti-PLEK (cat. no. A6305, ABclonal, dilution 1:200); Anti-LCP2 (cat. no. 12728-1-AP, Proteintech, dilution 1:200); Anti-HCK (cat. no. A2083, ABclonal, dilution 1:100); Anti-ITGAM (cat. no. A1581, ABclonal, dilution 1:100).

## Results

### Construction of WGCNA co-expression network

**Figure 1:**
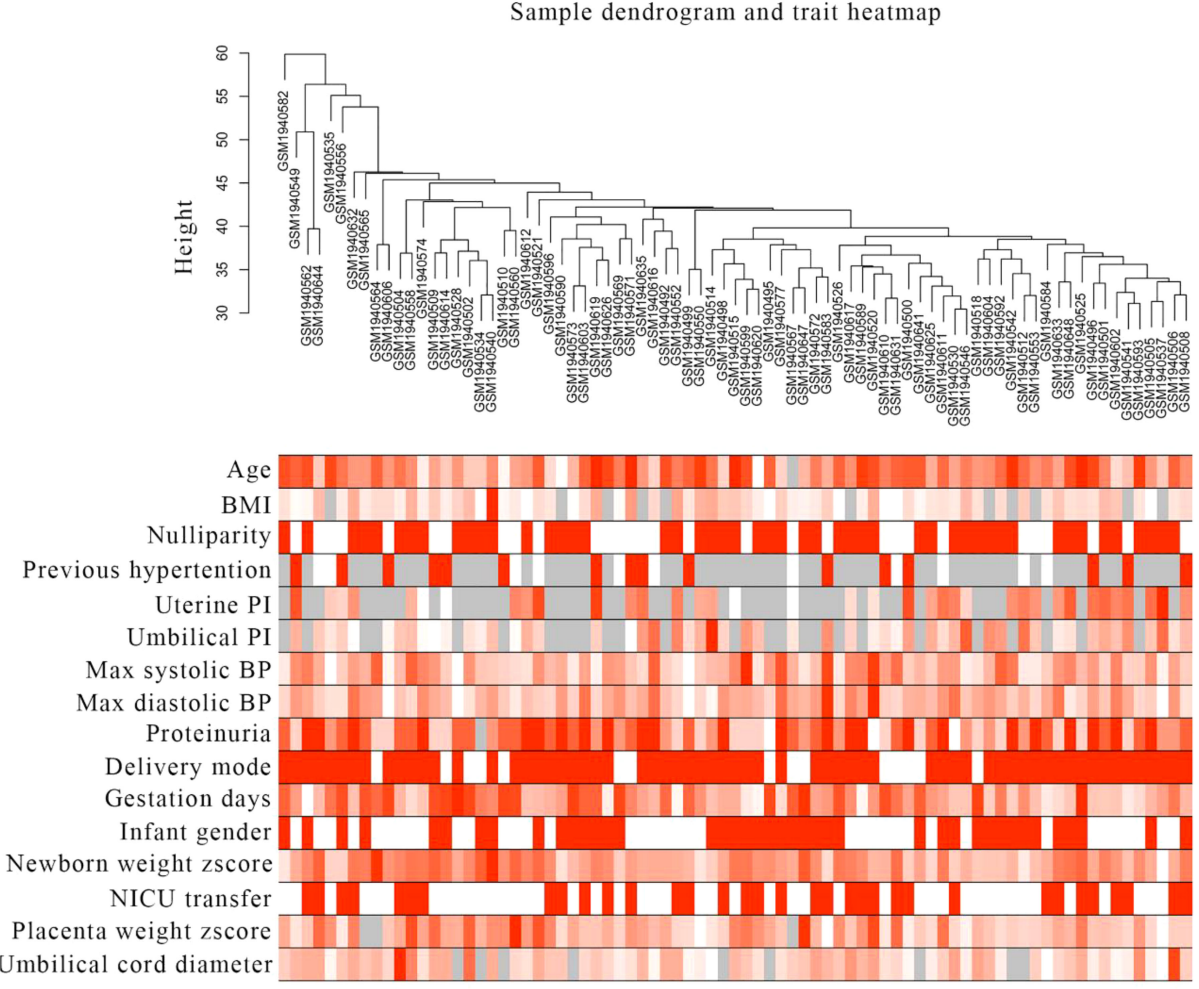
Sample clustering tree and clinical trait heat map in preeclampsia. The cut-off value was set as 60, and one outlier (GSM1940547) was found and removed. In this study, 16 clinical traits were described, including age, BMI, nulliparity, previous hypertension, mean uterine PI, mean umbilical PI, maximum systolic BP, maximum diastolic BP, proteinuria, delivery mode, gestation days, infant sex, newborn weight z-score, NICU transfer, placental weight z-score and umbilical cord diameter.

**Figure 2:**
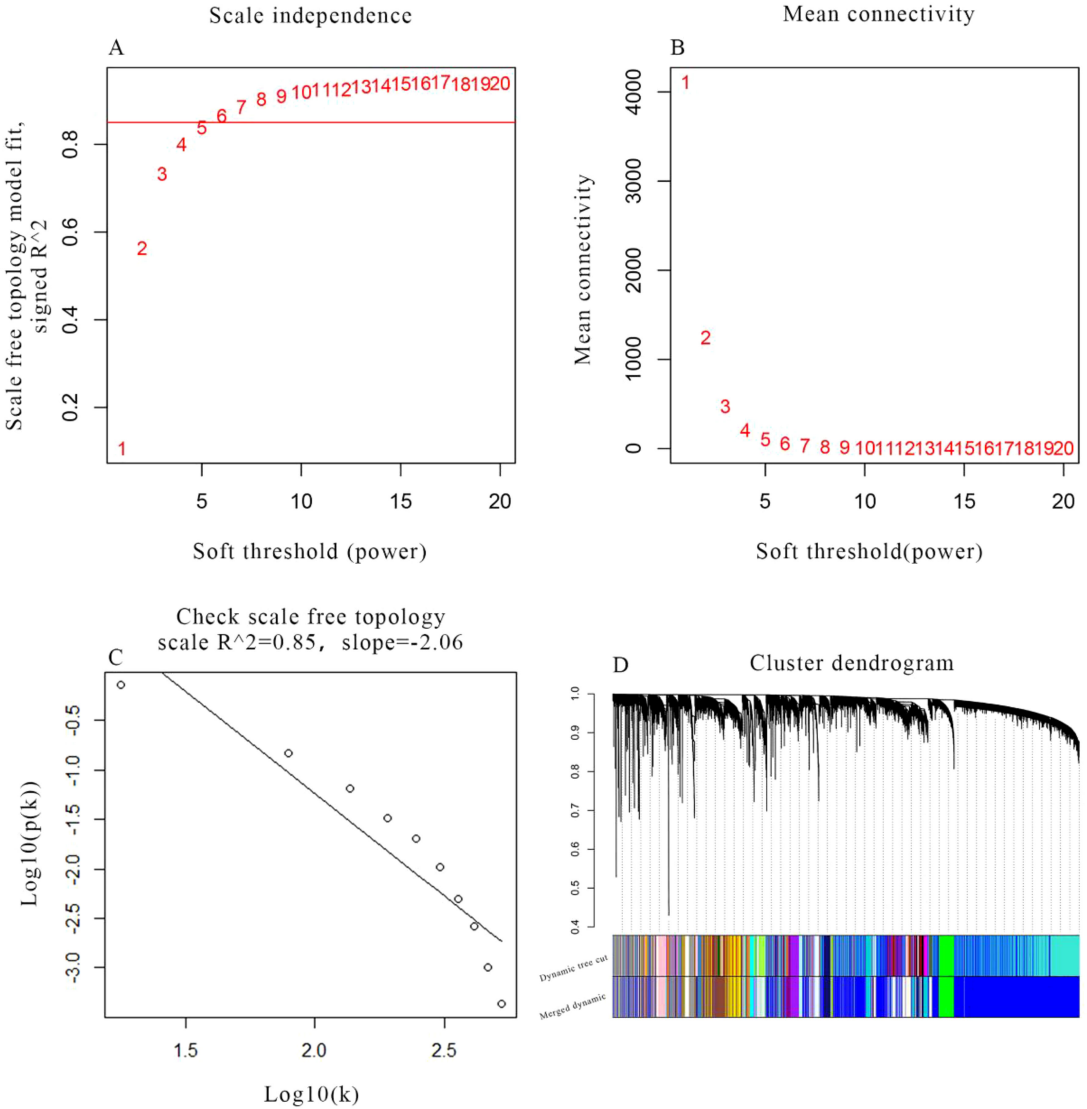
Selection of soft threshold power in WGCNA. **(A)** Scale independence and **(B)** mean connectivity of various soft-thresholding powers (β). **(C)** Scale-free topology when β=6. **(D)** Clustering tree map of GSE75010 gene. Each branch represents a gene, and the different colours below represent different co-expression modules.

After removing one outlier sample (GSM1940547), the remaining gene expression profiles and corresponding clinical traits of 79 preeclampsia samples from the GSE75010 dataset were utilized to construct the WGCNA network (**Figure 1**). A soft-thresholding power of β = 6 was selected to ensure optimal scale independence (>0.85) and mean connectivity (~0). The assessment of scale-free topology confirmed the appropriateness of the chosen soft-thresholding power, achieving a scale-free R² ≥ 0.85 (**Figure 2C**). Subsequently, 33 co-expression modules were identified, including modules named floral white, light green, maroon, thistle2, cyan, dark orange, pale turquoise, light pink4, plum2, light cyan, brown4, dark magenta, purple, Navajo white2, grey60, pale violet red3, sky blue3, ivory, pink, yellow-green, midnight blue, dark turquoise, light yellow, bisque4, white, salmon4, steel blue, dark slate blue, yellow, dark orange2, blue, and green. Genes without significant co-expression relationships were grouped into the grey module (**Figure 2D**).

### Identification of clinically relevant modules and core genes

**Figure 3:**
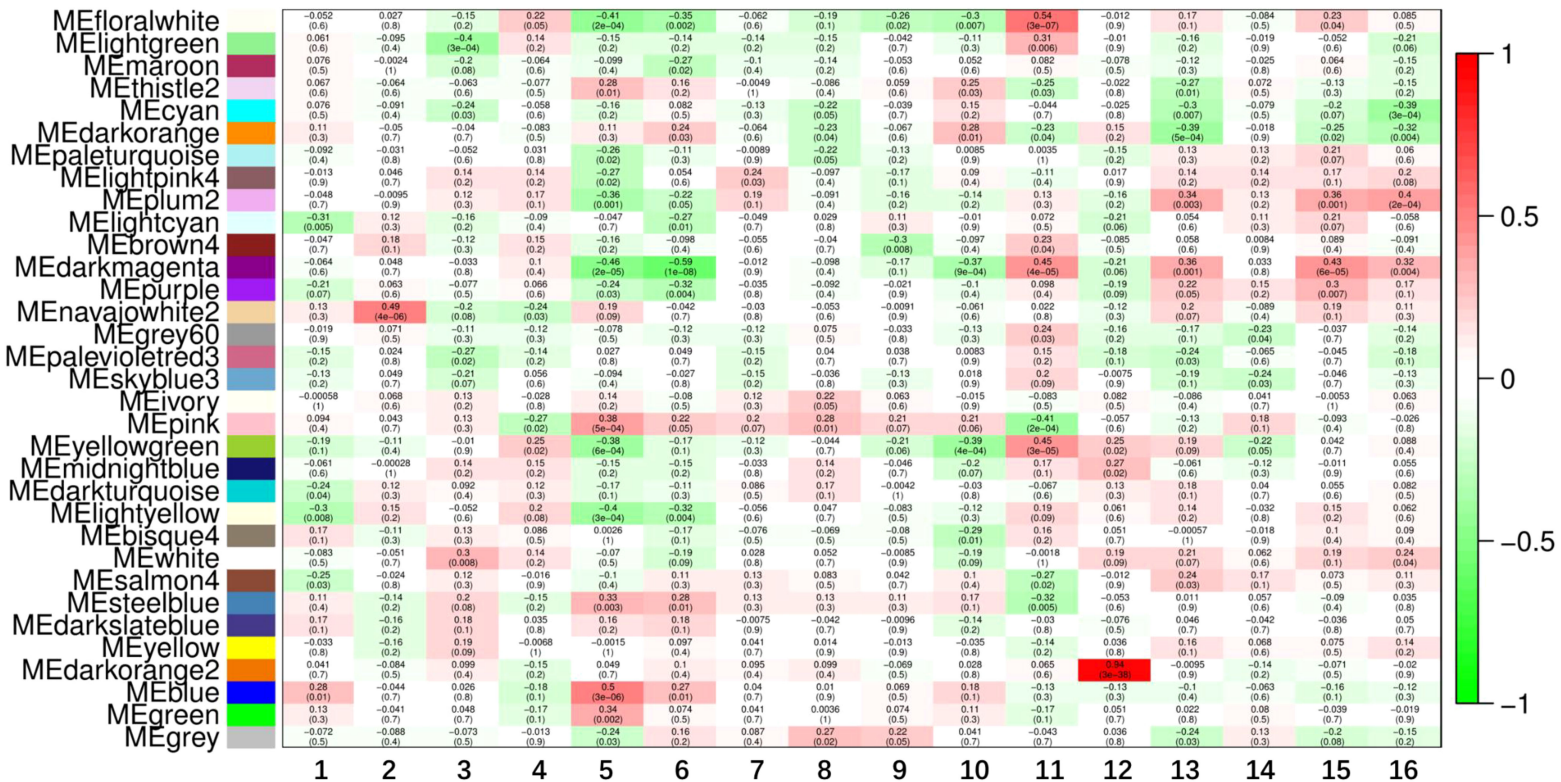
Identification of key modules. Heatmap of the correlation between module genes and clinical traits. Among them, the flower white module, plum2 module, deep magenta module and yellow-green module were closely related to a number of clinical traits (>3) and were regarded as important modules. 1, Age; 2. BMI; 3. Nulliparity; 4. Previous hypertension; 5. Uterine Pl; 6. Umbilical PI; 7. Max systolic BP; 8. Max diastolic BP; 9. Proteinuria; 10. Delivery mode; 11. Gestation days; 12. Infant gender; 13. Newborn weight zscore; 14. NICU transfer; 15. Placenta weight zscore; 16. Umbilical cord diameter.

To elucidate the associations between clinical traits in preeclampsia and gene modules, Pearson correlation analyses were conducted for each module against the clinical traits. Notable findings include: Light Green Module: Genes in this module exhibited significant associations with gestation duration (R=0.31, P=0.006) and nulliparity (R=-0.4, P=0.0003). Cyan Module: Genes in this module showed significant correlations with umbilical cord diameter (R=-0.39, P=0.0003). Dark Orange Module: Genes in this module were significantly associated with newborn weight z-score (R=-0.39, P=0.0005) and also with umbilical cord diameter (R=-0.32, P=0.004). Light Cyan Module: Genes in this module exhibited significant associations with age (R=-0.31, P=0.005). Navajo White2 Module: Genes in this module showed significant associations with BMI (R=0.49, P=0.000004). Purple Module: Genes in this module were significantly associated with mean umbilical PI (R=-0.32, P=0.004). Pink Module: Genes in this module were significantly associated with mean uterine PI (R=0.38, P=0.0005) and gestation days (R=-0.41, P=0.0002). Moreover, the Floral White, Plum2, Dark Magenta, and Yellow-Green modules demonstrated strong associations with multiple clinical traits (more than three), designating them as significant modules for further investigation (**Figure 3**). By evaluating the MM of each gene within these significant modules, a set of 75 genes with MM > 0.8 was identified as core genes of these modules (**Table 1**).

**Table 1:** Identification of module core genes.

Gene	Module color
PECAM1.1	dark magenta
GUCY1B3	dark magenta
CD34	dark magenta
GJC1	dark magenta
TCF4	dark magenta
PLSCR4	dark magenta
SPTAN1	dark magenta
MEF2C	dark magenta
FLI1	dark magenta
EGFLAM	dark magenta
KL	dark magenta
ADGRL2	dark magenta
PCDHB14	dark magenta
ADAMTSL3	dark magenta
CYTH4	floral white
BTK	floral white
CYBB	floral white
CD53	floral white
CD4	floral white
ITGAM	floral white
TFEC	floral white
HNMT	floral white
TBXAS1	floral white
MS4A14	floral white
MILR1	floral white
SLC7A7	floral white
SYK	floral white
TMSB4X	floral white
HCK	floral white
TYROBP	floral white
AOAH	floral white
CD84	floral white
NCKAP1L	floral white
CORO1A	floral white
CTSS	floral white
LRMP	floral white
CD86	floral white
TNFSF8	floral white
PIK3CG	floral white
TRPS1	floral white
LAIR1	floral white
PLEK	floral white
PRKCB	floral white
PTPRC	floral white
SH3BGRL	floral white
TLR1	floral white
TLR4	floral white
GPR65	floral white
C3AR1	floral white
FCER1G	floral white
AIF1	floral white
DOCK2	floral white
LCP2	floral white
LAPTM5	floral white
MYO1F	floral white
SAMHD1	floral white
TLR7	floral white
TLR8	floral white
ADAP2	floral white
ARHGAP15	floral white
RASSF4	floral white
HAVCR2	floral white
OLFML2B	plum2
SEC14L1	plum2
AFF2	plum2
HIP1.1	plum2
KCNK3	plum2
PLCG1	plum2
TMEM204	plum2
ATP1A1	yellow green
ZMYND11	yellow green
TMEM139	yellow green
THSD4	yellow green
FBN2	yellow green
SLC23A2	yellow green

Annotation: Genes in significant modules with MM ≥ 0.8 were set as module core genes and showed in the list.

### Functional enrichment and KEGG pathway analysis for module core genes

**Figure 4:**
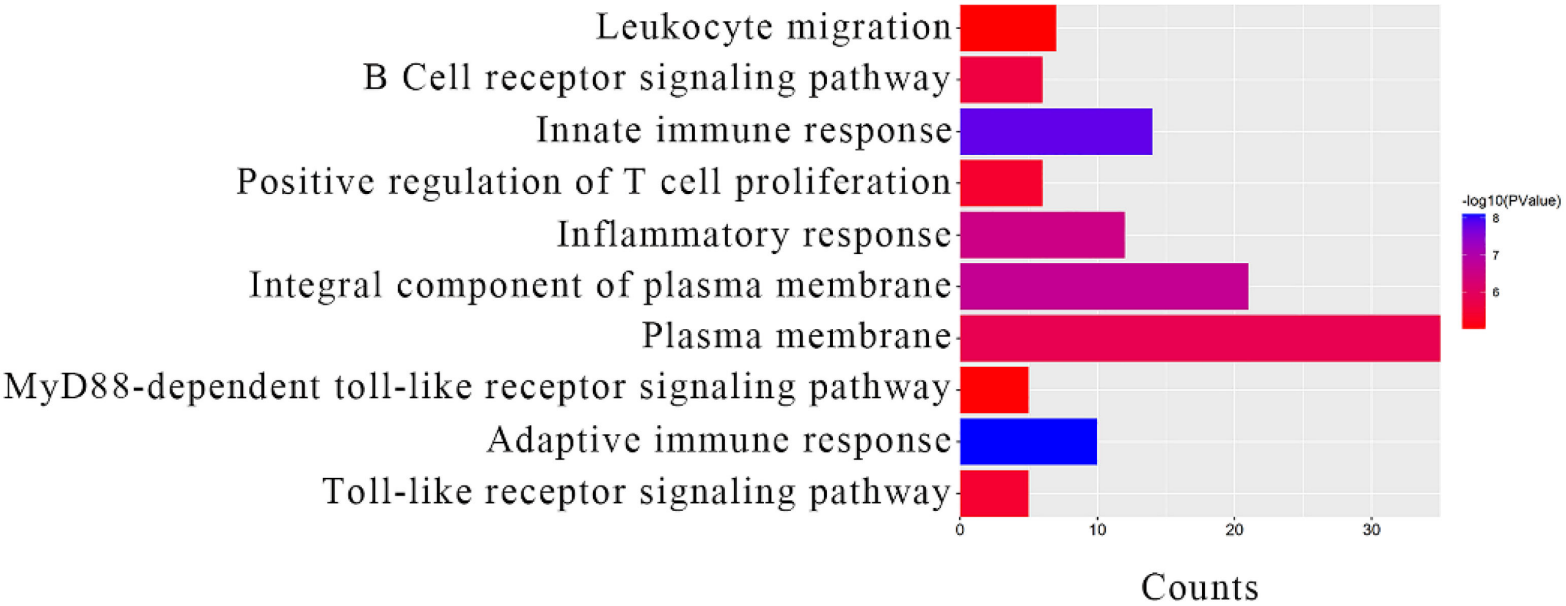
GO analysis results of module core genes.

The module core genes were submitted to the DAVID database for GO functional annotation. The top 10 results revealed significant enrichments in key biological processes and cellular components for the genes within clinically important modules: Adaptive immune response (BP, GO: 0002250); Innate immune response (BP, GO: 0045087); Integral component of plasma membrane (CC, GO: 0005887); Inflammatory response (BP, GO: 0006954); Plasma membrane (CC, GO: 0005886); B cell receptor signaling pathway (BP, GO: 0050853); Toll-like receptor signaling pathway (BP, GO: 0002224); Positive regulation of T cell proliferation (BP, GO: 0042102); MyD88-dependent Toll-like receptor signaling pathway (BP, GO: 0002755); Leukocyte migration (BP, GO: 0050900) (**Figure 4**). The KEGG pathway enrichment analysis highlighted that the module core genes were significantly enriched in pathways associated with: Platelet activation

**Figure 5:**
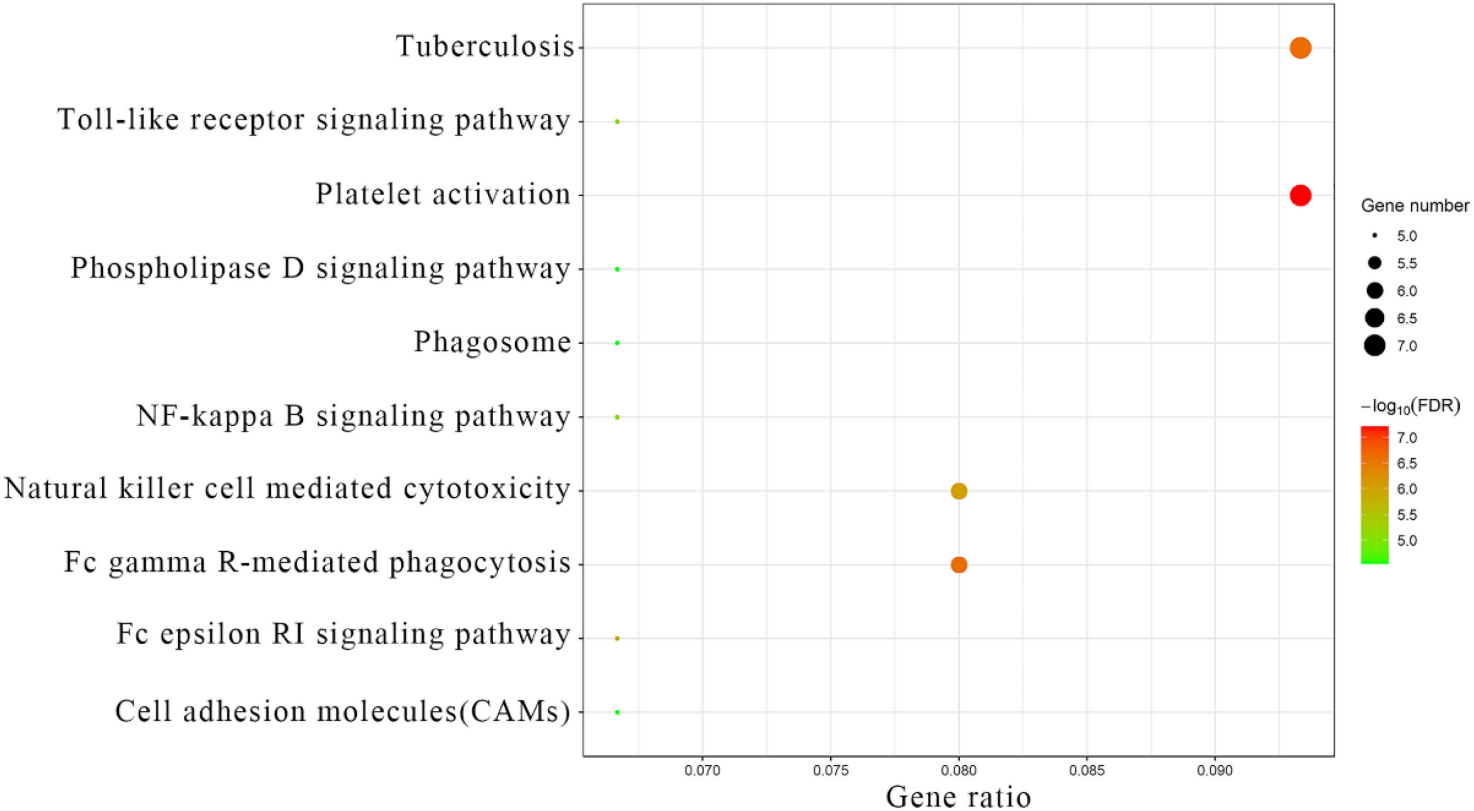
KEGG analysis results of module core genes.

Fc gamma R-mediated phagocytosis; Tuberculosis; Natural killer cell-mediated cytotoxicity; Fc epsilon RI signaling pathway; NF-kappa B signaling; Toll-like receptor signaling; Cell adhesion molecules; Phospholipase D signaling; Phagosomes (**Figure 5**). These analyses provide insights into the functional roles and pathways related to the module core genes, shedding light on their potential involvement in the pathogenesis and progression of preeclampsia.

### Differential expression analysis of core genes in preeclampsia placentas

**Figure 6:**
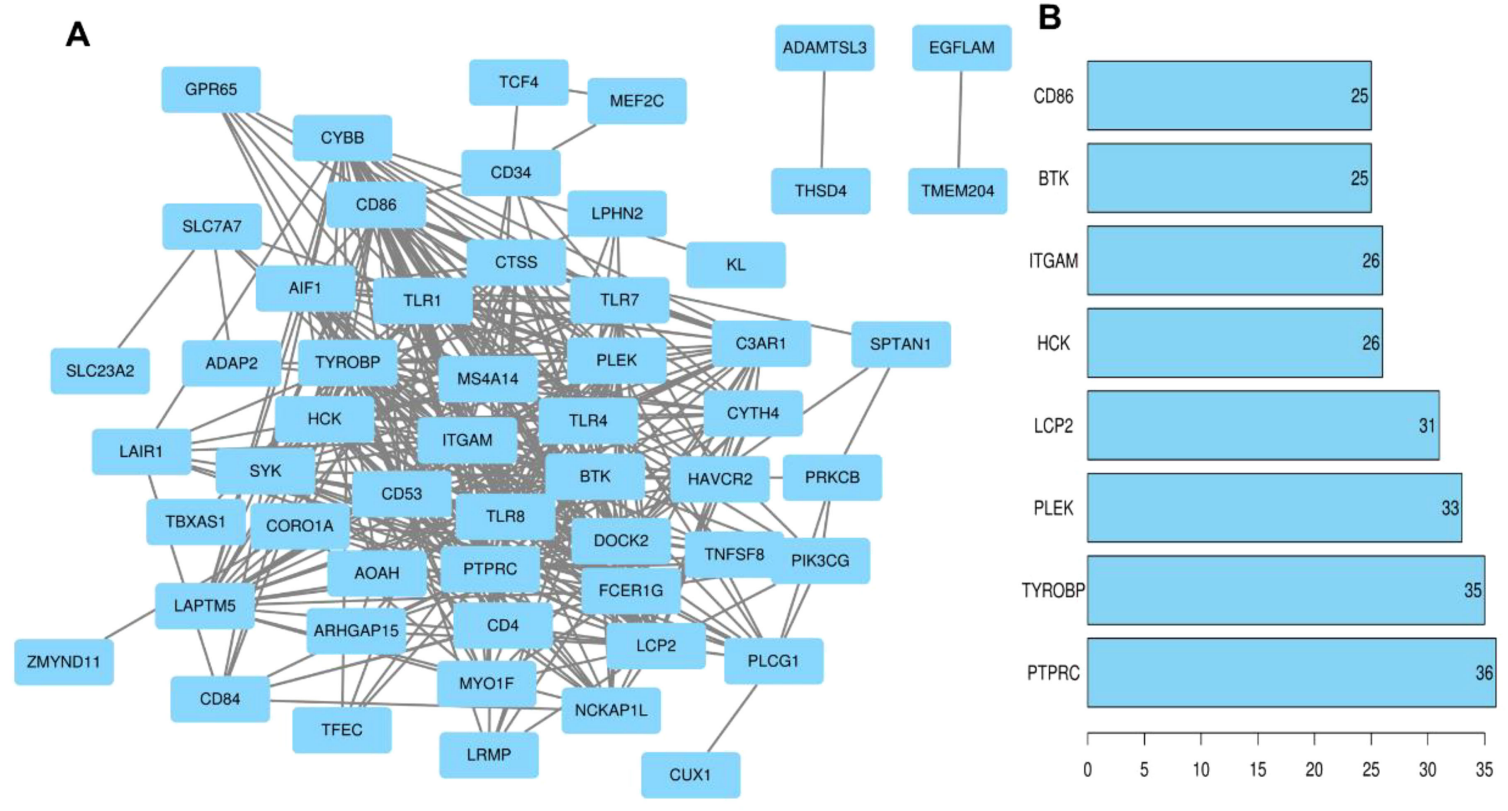
PPI network construction and selection of hub genes. **(A)** Flower white, plum2, dark magenta and yellow-green module genes for PPI network construction. **(B)** Genes with the top 10% of scores based on number of connections.

**Figure 7:**
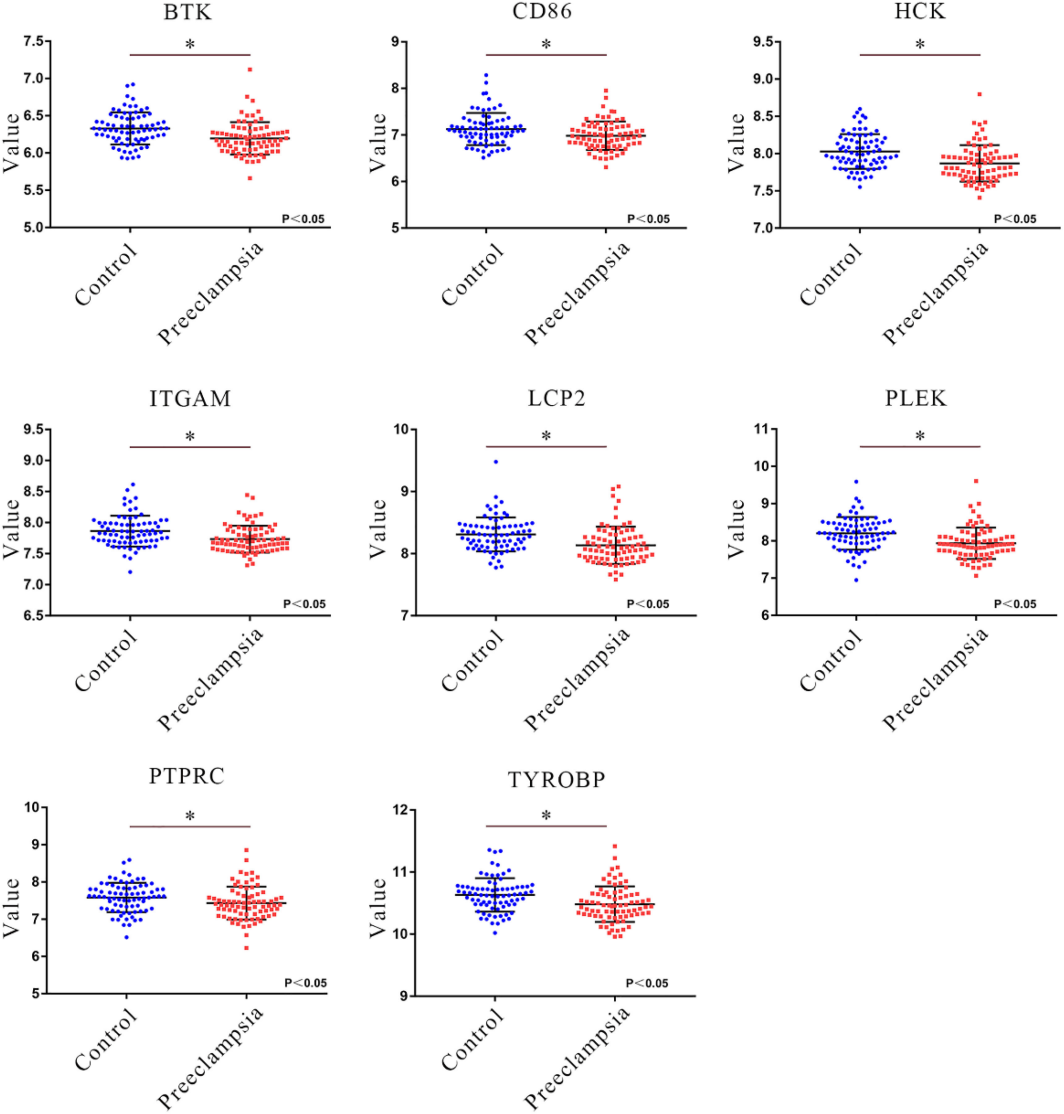
Verification of hub gene expression. Comparison of the expression levels of core genes (*PTPRC, TYROBP, PLEK, LCP2, HCK, ITGAM, BTK, CD86*) in the GSE75010 dataset in the preeclampsia and control groups. Blue squares represent the gene expression levels in the control group (n=77), and red squares represent the gene expression levels in the preeclampsia group (n=79). *P<0.05.

The module core genes were used to construct a PPI network via the String database (**Figure 6A**). Using R, gene connectivity was calculated, identifying those with degree scores in the top 10%, such as *PTPRC, TYROBP, PLEK, LCP2, HCK, ITGAM, BTK*, and *CD86*, as hub genes (**Figure 6B**). The expression patterns of these core genes were compared between preeclampsia placentas and non-preeclampsia placentas. Notably, in the GSE75010 dataset, the expression levels of the identified hub genes were significantly downregulated in preeclampsia placentas compared to non-preeclampsia placentas (**Figure 7**).

**Figure 8:**
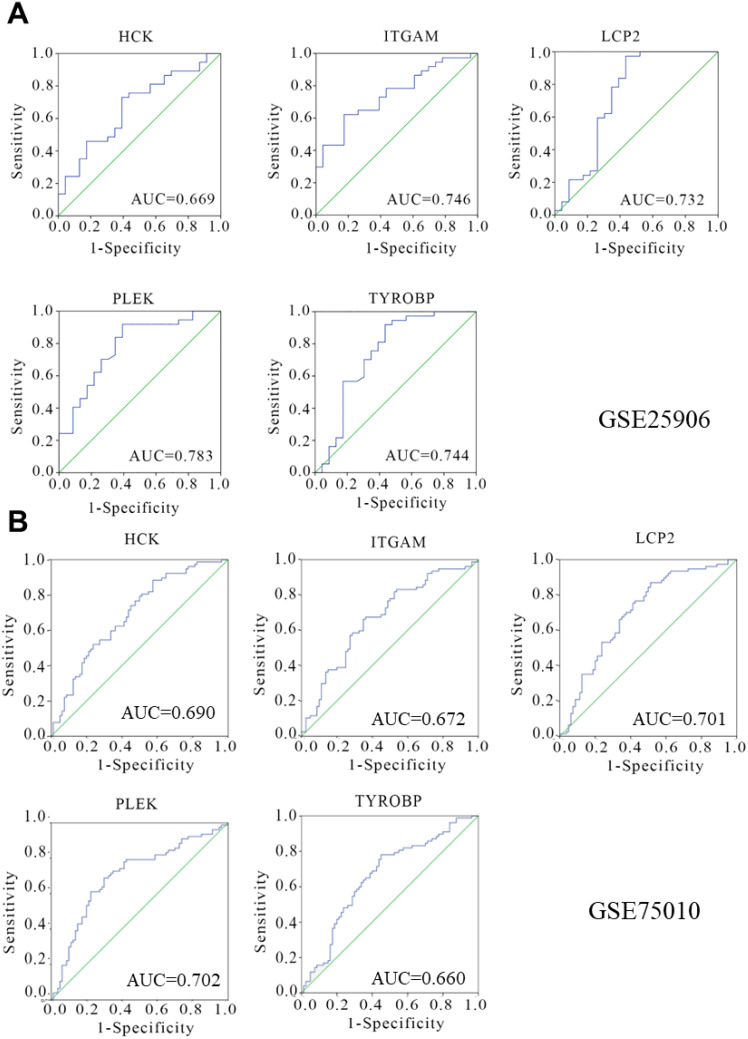
Diagnostic efficiency verification of hub genes. The expression of hub genes in the GSE25906 dataset **(A)** and GSE75010 dataset **(B)** was obtained, and a receiver operating curve was constructed to verify the diagnostic value. The 5 genes (*TYROBP, PLEK, LCP2, HCK, ITGAM*) shown in the figure have significant diagnostic efficiency.

Receiver Operating Characteristic (ROC) curves were used to evaluate the diagnostic potential of the hub gene expression profile. For GSE25906 dataset, which included 23 preeclampsia placentas and 37 non-preeclampsia placentas, The analysis demonstrated that *TYROBP* (AUC=0.744), *PLEK* (AUC=0.783), *LCP2* (AUC=0.732), *HCK* (AUC=0.669), and *ITGAM* (AUC=0.746) exhibited notable diagnostic efficacy (**Figure 8A**). Consistently, for GSE75010 dataset, the analysis demonstrated that *TYROBP* (AUC=0.660), *PLEK* (AUC=0.702), *LCP2* (AUC=0.701), *HCK* (AUC=0.690), and *ITGAM* (AUC=0.672) (**Figure 8B**). This analysis supports the diagnostic relevance of these genes in preeclampsia.

### GSEA and immunohistochemical validation of hub genes

To explore the potential biological roles of the identified hub genes in preeclampsia, the GSE75010 dataset was utilized for GSEA. The findings revealed that these five hub genes were collectively enriched across 17 pathways, including T cell receptor signaling, leukocyte transendothelial migration, viral myocarditis, primary immunodeficiency, FC epsilon RI signaling, prion diseases, cell adhesion molecules, natural killer cell-mediated cytotoxicity, complement and coagulation cascades, leishmania infection, systemic lupus erythematosus, antigen processing and presentation, intestinal immune network for IgA production, pantothenate and CoA biosynthesis, NOD-like receptor signaling, pathogenic Escherichia coli infection, and glutathione metabolism (**Table 2**).

**Table 2:** Pathways enriched in 5 genes in GSEA analysis.

Gene Pathways	*HCK*	*ITGAM*	*LCP2*	*PLEK*	*TYROBP*
KEGG_T_CELL_RECEPTOR_SIGNALING_PATHWAY	2.12	2.01	1.82	1.83	1.9
KEGG_LEUKOCYTE_TRANSENDOTHELIAL_MIGRATION	1.99	2.05	1.81	1.75	1.97
KEGG_VIRAL_MYOCARDITIS	1.88	1.71	1.62	1.62	1.72
KEGG_PRIMARY_IMMUNODEFICIENCY	1.88	1.76	1.61	1.86	1.94
KEGG_FC_EPSILON_RI_SIGNALING_PATHWAY	1.88	1.89	1.69	1.89	1.81
KEGG_PRION_DISEASES	1.86	1.76	1.76	1.81	1.83
KEGG_CELL_ADHESION_MOLECULES_CAMS	1.86	1.86	1.65	1.77	1.79
KEGG_NATURAL_KILLER_CELL_MEDIATED_CYTOTOXICITY	1.84	1.73	1.52	1.62	1.8
KEGG_COMPLEMENT_AND_COAGULATION_CASCADES	1.81	1.71	1.58	1.72	1.79
KEGG_LEISHMANIA_INFECTION	1.8	1.7	1.64	1.69	1.67
KEGG_SYSTEMIC_LUPUS_ERYTHEMATOSUS	1.77	1.68	1.65	1.67	1.62
KEGG_ANTIGEN_PROCESSING_AND_PRESENTATION	1.68	1.66	1.53	1.57	1.63
KEGG_INTESTINAL_IMMUNE_NETWORK_FOR_IGA_PRODUCTION	1.67	1.52	1.52	1.56	1.58
KEGG_PANTOTHENATE_AND_COA_BIOSYNTHESIS	1.66	1.52	1.53	1.55	1.69
KEGG_NOD_LIKE_RECEPTOR_SIGNALING_PATHWAY	1.63	1.65	1.61	1.74	1.63
KEGG_PATHOGENIC_ESCHERICHIA_COLI_INFECTION	1.61	1.88	1.57	1.68	1.64
KEGG_GLUTATHIONE_METABOLISM	1.59	1.61	1.53	1.78	1.53

Annotation: The GSE75010 was used for GSEA analysis. The values in the table represent the NES value. The table listed KEGG pathways in which all genes satisfy P<0.05. The number in units means the NES value of each for the pathway.

**Figure 9:**
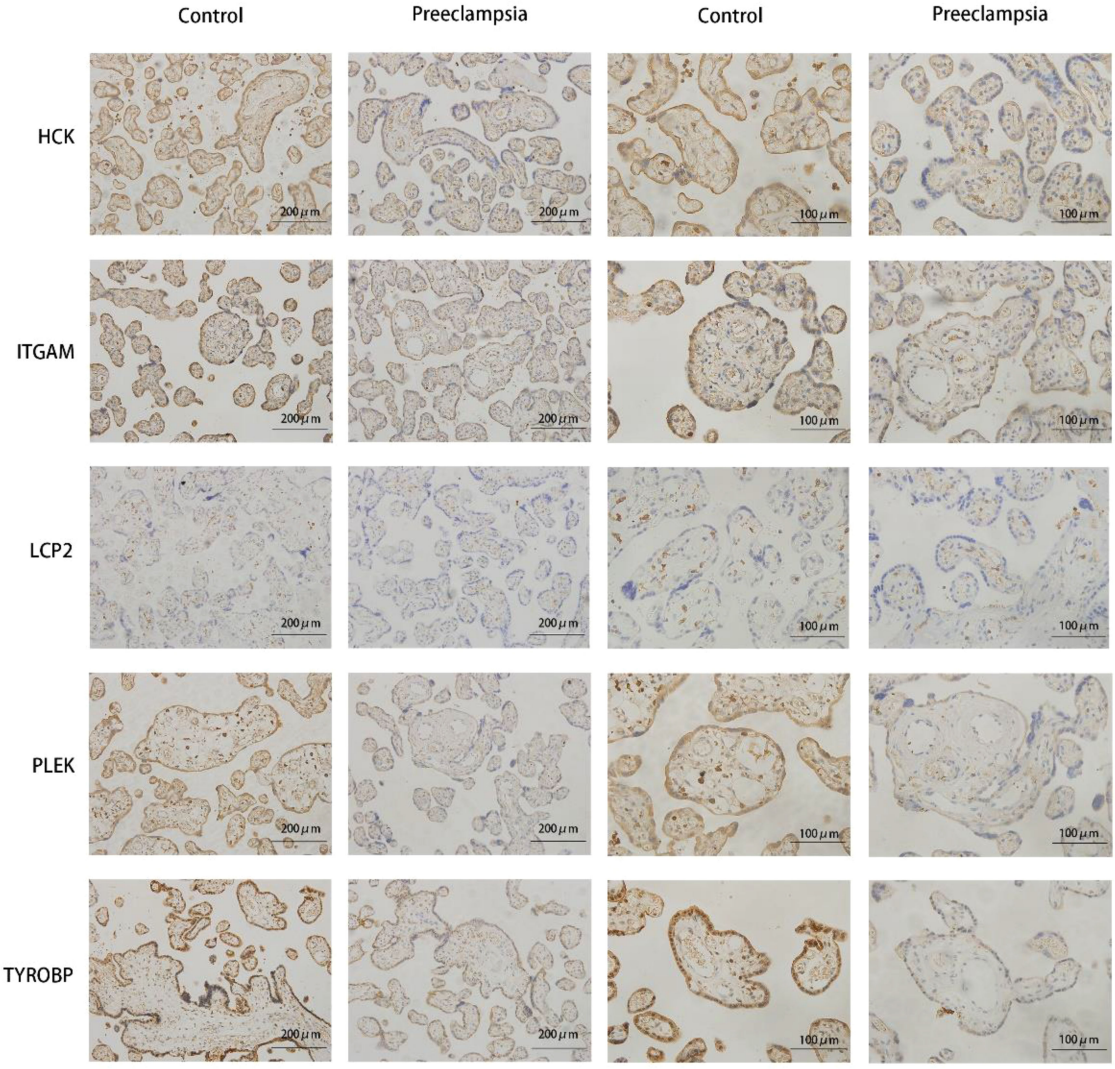
Validation results of immunohistochemical staining of hub genes. Representative images of immunohistochemical staining of the TYROBP, PLEK, LCP2, HCK, and ITGAM genes in the placental tissues of controls and preeclampsia patients.

To validate the differential expression of hub genes between the two cohorts, immunohistochemistry assays were conducted on 18 preeclamptic placental tissues and 15 normal control placental tissues. Notably, there were no significant differences in gestational age or BMI between the two groups of expectant mothers (**Table 3**). The outcomes of the immunohistochemical analysis revealed lower expression levels of these hub genes (*TYROBP, PLEK, LCP2, HCK, ITGAM*) in the preeclampsia group compared to the normal group (**Figure 9**). These findings provide further evidence for the involvement of these hub genes in the pathogenesis of preeclampsia and underscore their potential as key regulators in this complex disorder.

**Table 3:** Clinical characteristics of two groups of pregnant women.

Clinical traits	Group	
	Control (n=15)	Preeclampsia (n=18)
Gestational age, weeks	37.20 ± 2.66	35.99 ± 3.30
Birth weight, g	2847.33 ± 603.83	2247.78 ± 749.61*
Placenta weight, g	584.67 ± 106.90	493.33 ± 139.58*
Systolic pressure, mm Hg	111.67 ± 7.67	163.67 ± 13.58*
Diastolic pressure, mm Hg	74.67 ± 4.05	102.78 ± 9.37*
Maternal BMI, kg/m^2^	26.13 ± 2.40	27.45 ± 2.70
Proteinuria, (-,+,++,+++)	15-0-0-0	0-15-3-0***

Annotation: Clinical characteristics of two groups of pregnant women for immunohistochemical staining. The values were expressed as mean ± SD. The P value between the preeclampsia group and the control group was given. *P<0.05, ***P<0.001.

## Discussion

Preeclampsia is one of the most serious pregnancy complications, posing significant risks to both maternal and fetal health. Extensive research into the pathogenesis of preeclampsia has identified various contributing factors, including genetic influences, angiogenic imbalances, immune maladaptation, disrupted lipid metabolism, impaired trophoblast invasion, increased cell death, aberrant placental development, and heightened maternal inflammation. Notably, most studies suggest that irregularities in placental formation and alterations in the placental gene expression profile play crucial roles in compromising trophoblast migration and invasion, ultimately leading to the onset of preeclampsia (12). Exploring changes in the placental gene profile within the context of preeclampsia is of paramount importance. Recent work by Kang et al. employed Robust Rank Aggregation (RRA) to analyze differentially expressed genes, subsequently using methodologies such as WGCNA to identify genes potentially linked to preeclampsia onset (13). While Kang’s WGCNA approach incorporated three clinical traits, our investigation encompassed a broader array of clinical information, utilizing the entire dataset to establish the WGCNA framework. It is essential to note that even genes that do not show significant differences in differential expression analyses cannot be dismissed as irrelevant to the disease process.

In this study, we retrieved datasets GSE75010 (discovery cohort) and GSE25906 (validation cohort) from the GEO database. Through WGCNA, GO analysis, KEGG analysis, PPI network construction, expression profiling, diagnostic validation, and immunohistochemistry (IHC) experiments, we identified *TYROBP, PLEK, LCP2, HCK*, and *ITGAM* as central hub genes potentially implicated in the pathogenesis of preeclampsia. These findings contribute to a deeper understanding of the molecular mechanisms underlying preeclampsia and may pave the way for novel diagnostic and therapeutic strategies targeting these key genes.

In the study by Awamleh (14), it was demonstrated that *ITGAM* mRNA expression is reduced in the placentas of patients with preeclampsia and intrauterine growth restriction. Furthermore, it was noted that *ITGAM* is regulated by miR-210-5p and contributes to the pathogenesis of preeclampsia by inhibiting the invasion and migration of HTR8 cells. Our current investigation identified the ITGAM gene within the floral white module, revealing a negative correlation with the mean uterine PI and mean umbilical PI, while showing a positive correlation with the total days of pregnancy. Additionally, through immunohistochemistry and expression analysis, we observed that ITGAM expression is diminished in preeclampsia and holds promising diagnostic value. Consequently, we postulate that ITGAM serves as a protective factor in preeclampsia, consistent with prior research findings.

TYROBP encodes a transmembrane signal peptide that contains a tyrosine-based immunoreceptor activation motif (ITAM) (15). Identified as a universal marker for macrophages in both mouse and human tissues (16), TYROBP is associated with the maternal innate immune response to proinflammatory stimuli from the placenta, a hallmark of preeclampsia pathogenesis. LCP2 encodes a substrate that activates the protein tyrosine kinase pathway via the T cell antigen receptor (17). Notably, reduced LCP2 protein expression has been observed in the colonic epithelial cells of a spontaneously hypertensive rat model (18). Studies by Pan (19) suggested a potential link between TYROBP and LCP2 and the development of atherosclerosis in a high-fat diet Tibetan minipig model. Furthermore, PLEK, as discovered by Song et al., is implicated in interactions with IRF-8, contributing to periodontal inflammation based on expression profile analyses of periodontitis and normal gingival tissues, indicating its potential involvement in inflammatory responses (20). PLEK has also been associated with the pathogenesis of abdominal aortic aneurysms, a vascular disease possibly linked to oxidative stress and inflammation (21). Lastly, HCK, a member of the Src family of protein kinases, participates in various signal transduction pathways that regulate cell functions such as growth, proliferation, differentiation, migration, and apoptosis (22). In colorectal tumors, HCK is linked to prognosis and immune cell responses during local inflammation (23).

While these genes are recognized for their roles in inflammatory responses, immune reactions, cell migration, and invasion, their association with preeclampsia has not been extensively reported. Our research identified these genes within the floral white module, revealing a negative correlation with the mean uterine PI and mean umbilical PI, as well as a positive correlation with the total days of pregnancy, indicating substantial diagnostic value. Furthermore, gene expression analyses and immunohistochemistry results indicated lower placental expression levels of these genes in preeclampsia patients compared to controls. Hence, we speculate that TYROBP, PLEK, LCP2, and HCK may serve as protective factors in preeclampsia.

However, it is important to acknowledge the limitations of our study. One of the limitations is the relatively small sample size used in this research, which may impact the generalizability of the findings. Additionally, the study focused on a specific population group, limiting the broader applicability of the results to a more diverse population. Furthermore, the cross-sectional nature of the study design restricts our ability to establish causal relationships between the identified genes and the progression of preeclampsia. Future longitudinal studies with larger and more diverse cohorts are needed to validate our findings and further elucidate the potential roles of these genes in the pathogenesis of preeclampsia. Additionally, it is difficult to conclude whether these genes actually play a causal role in the development of preeclampsia or are merely correlated with the condition. Moreover, many factors such as diet, stress, environment and even may aggravate preeclampsia associated with these genes.

Despite these limitations, our study provides valuable insights into the potential protective role of the identified genes in preeclampsia and sets the stage for future research in this area. Addressing these limitations in future studies will contribute to a more comprehensive understanding of the molecular mechanisms underlying preeclampsia and may lead to the development of novel diagnostic and therapeutic approaches for this complex disorder.

In conclusion, through WGCNA, GO analysis, KEGG analysis, expression profiling, diagnostic value assessments, GSEA, and immunohistochemical staining, our study has identified five genes (*TYROBP, PLEK, LCP2, HCK, ITGAM*) that may play a protective role in the progression of preeclampsia. These genes show promise for improving diagnostic and treatment strategies for this condition.

## Data Availability

The datasets presented in this study can be found in online repositories. The names of the repository/repositories and accession number(s) can be found in the article/supplementary material.

## References

[1] Rana S Lemoine E Granger JP Karumanchi SA. Preeclampsia: pathophysiology, challenges, and perspectives. Circ Res. (2019) 124:1094–112. doi: 10.1161/CIRCRESAHA.118.313276 30920918

[2] Alsnes IV Vatten LJ Fraser A Bjørngaard JH Rich-Edwards J Romundstad PR. Hypertension in pregnancy and offspring cardiovascular risk in young adulthood: prospective and sibling studies in the HUNT study (Nord-trøndelag health study) in Norway. Hypertension. (2017) 69:591–8. doi: 10.1161/HYPERTENSIONAHA.116.08414 28223467

[3] Gumusoglu SB Chilukuri ASS Santillan DA Santillan MK Stevens HE. Neurodevelopmental outcomes of prenatal preeclampsia exposure. Trends Neurosci. (2020) 43:253–68. doi: 10.1016/j.tins.2020.02.003 PMC7170230 32209456

[4] Raguema N Moustadraf S Bertagnolli M. Immune and apoptosis mechanisms regulating placental development and vascularization in preeclampsia. Front Physiol. (2020) 11:98. doi: 10.3389/fphys.2020.00098 32116801 PMC7026478

[5] Lei D Deng N Wang S Huang J Fan C. Upregulated ARRDC3 limits trophoblast cell invasion and tube formation and is associated with preeclampsia. Placenta. (2020) 89:10–9. doi: 10.1016/j.placenta.2019.10.009 31665660

[6] Langfelder P Horvath S. WGCNA: an R package for weighted correlation network analysis. BMC Bioinf. (2008) 9:559. doi: 10.1186/1471-2105-9-559 PMC2631488 19114008

[7] Meng Y Li C Liu CX. Immune cell infiltration landscape and immune marker molecular typing in preeclampsia. Bioengineered. (2021) 12:540–54. doi: 10.1080/21655979.2021.1875707 PMC8806319 33535891

[8] Edgar R Domrachev M Lash AE. Gene Expression Omnibus: NCBI gene expression and hybridization array data repository. Nucleic Acids Res. (2002) 30:207–10. doi: 10.1093/nar/30.1.207 PMC99122 11752295

[9] Huang da W Sherman BT Lempicki RA. Systematic and integrative analysis of large gene lists using DAVID bioinformatics resources. Nat Protoc. (2009) 4:44–57. doi: 10.1038/nprot.2008.211 19131956

[10] Wu J Mao X Cai T Luo J Wei L. KOBAS server: a web-based platform for automated annotation and pathway identification. Nucleic Acids Res. (2006) 34:W720–4. doi: 10.1093/nar/gkl167 PMC1538915 16845106

[11] Szklarczyk D Gable AL Lyon D Junge A Wyder S Huerta-Cepas J. STRING v11: protein-protein association networks with increased coverage, supporting functional discovery in genome-wide experimental datasets. Nucleic Acids Res. (2019) 47:D607–d613. doi: 10.1093/nar/gky1131 30476243 PMC6323986

[12] Mohamad MA Mohd Manzor NF Zulkifli NF Zainal N Hayati AR Ahmad Asnawi AW. A review of candidate genes and pathways in preeclampsia-an integrated bioinformatical analysis. Biology. (2020) 9(1-19). doi: 10.3390/biology9040062 PMC7235730 32230784

[13] Kang Q Li W Xiao J Yu N Fan L Sha M. Integrated analysis of multiple microarray studies to identify novel gene signatures in preeclampsia. Placenta. (2021) 105:104–18. doi: 10.1016/j.placenta.2021.01.023 33571845

[14] Awamleh Z Han VKM. Identification of miR-210-5p in human placentae from pregnancies complicated by preeclampsia and intrauterine growth restriction, and its potential role in the pregnancy complications. Pregnancy Hypertens. (2020) 19:159–68. doi: 10.1016/j.preghy.2020.01.002 32014817

[15] Ascone G Di Ceglie I Walgreen B Sloetjes AW Lindhout E Bot I. High LDL levels lessen bone destruction during antigen-induced arthritis by inhibiting osteoclast formation and function. Bone. (2020) 130:115140. doi: 10.1016/j.bone.2019.115140 31712132

[16] Dang D Taheri S Das S Ghosh P Prince LS Sahoo D. Computational approach to identifying universal macrophage biomarkers. Front Physiol. (2020) 11:275. doi: 10.3389/fphys.2020.00275 32322218 PMC7156600

[17] Wang X Li JP Chiu LL Lan JL Chen DY Boomer J. Attenuation of T cell receptor signaling by serine phosphorylation-mediated lysine 30 ubiquitination of SLP-76 protein. J Biol Chem. (2012) 287:34091–100. doi: 10.1074/jbc.M112.371062 PMC3464518 22902619

[18] Yang T Li H Oliveira AC Goel R Richards EM Pepine CJ. Transcriptomic signature of gut microbiome-contacting cells in colon of spontaneously hypertensive rats. Physiol Genomics. (2020) 52:121–32. doi: 10.1152/physiolgenomics.00087.2019 PMC7099409 31869283

[19] Pan Y Yu C Huang J Rong Y Chen J Chen M. Bioinformatics analysis of vascular RNA-seq data revealed hub genes and pathways in a novel Tibetan minipig atherosclerosis model induced by a high fat/cholesterol diet. Lipids Health Dis. (2020) 19:54. doi: 10.1186/s12944-020-01222-w 32213192 PMC7098151

[20] Song L Yao J He Z Xu B. Genes related to inflammation and bone loss process in periodontitis suggested by bioinformatics methods. BMC Oral Health. (2015) 15:105. doi: 10.1186/s12903-015-0086-7 26334995 PMC4559289

[21] Hinterseher I Erdman R Elmore JR Stahl E Pahl MC Derr K. Novel pathways in the pathobiology of human abdominal aortic aneurysms. Pathobiology. (2013) 80:1–10. doi: 10.1159/000339303 22797469 PMC3782105

[22] Wang J Chen R Liu X Shen J Yan Y Gao Y. Hck promotes neuronal apoptosis following intracerebral hemorrhage. Cell Mol Neurobiol. (2017) 37:251–61. doi: 10.1007/s10571-016-0365-0 PMC11482238 27053350

[23] Roseweir AK Powell A Horstman SL Inthagard J Park JH McMillan DC. Src family kinases, HCK and FGR, associate with local inflammation and tumour progression in colorectal cancer. Cell Signal. (2019) 56:15–22. doi: 10.1016/j.cellsig.2019.01.007 30684564

